# Biogeographic patterns of aerobic anoxygenic phototrophic bacteria reveal an ecological consistency of phylogenetic clades in different oceanic biomes

**DOI:** 10.1038/s41598-018-22413-7

**Published:** 2018-03-07

**Authors:** Anne-Catherine Lehours, François Enault, Dominique Boeuf, Christian Jeanthon

**Affiliations:** 1 Université Clermont Auvergne, CNRS, Laboratoire Microorganismes: Génome et Environnement, F-63000 Clermont-Ferrand, France; 2 Daniel K. Inouye Center for Microbial Oceanography Research and Education (C-MORE), University of Hawaii at Manoa, Honolulu, HI 96822 USA; 3 CNRS, Sorbonne Université, Station Biologique de Roscoff, Adaptation et Diversité en Milieu Marin, F-29680 Roscoff, France

## Abstract

In marine environments, aerobic anoxygenic phototrophic (AAP) bacterial assemblages vary in space and along environmental gradients but the factors shaping their diversity and distribution at different taxonomic levels remain poorly identified. Using sets of sequences encoding the *M* sub-unit of the photosynthetic apparatus from different oceanic regions, we prioritized the processes underlying AAP bacterial biogeographical patterns. The present analysis offers novel insights into the ecological distribution of marine AAP bacteria and highlights that physiological constraints play a key role in structuring AAP bacterial assemblages at a global scale. Salinity especially seems to favor lineage-specific adaptations. Moreover, by inferring the evolutionary history of habitat transitions, a substantial congruence between habitat and evolutionary relatedness was highlighted. The identification of ecological cohesive clades for AAP bacteria suggests that prediction of AAP bacterial assemblages is possible from marine habitat properties.

## Introduction

Anoxygenic phototrophic bacteria were long considered to be ecological oddities from specialized habitats^1^ limiting their relevance for the biosphere. This view has been challenged when bacteriochlorophyll *a* (Bchl *a*), the primary pigment of anoxygenic photosynthesis, was found to be distributed in surface waters of the open ocean^2^. This finding attracted a substantial scientific interest because the light-based metabolic strategy of aerobic anoxygenic phototrophic (AAP) bacteria implied a possible significant revision of oceanic energy budget^2^. However, the ecology of AAP bacteria is still far from being understood^3^. Unlike classical photosynthesis, the light energy harvested by AAP bacteria does not fuel the CO_2_ fixation as in autotrophic cells^4^. Genomic and physiological evidences showed that they are photoheterotrophic bacteria, using both organic substrates and light for their carbon and energy requirements^5^. This puzzling life style challenged the classical view of bacteria being dependent on recycling dissolved organic matter and raised questions about the selective advantage of phototrophy for heterotrophic bacterial communities. From an ecological view, the parsimonious explanation was that photoheterotrophy enables microbes to survive adverse conditions and/or to outgrow competitors^6^. It was therefore postulated that the ability to use light may be especially beneficial in nutrient-poor marine environments^2^. But the hypothesis was repeatedly disproved as AAP bacteria are more abundant in productive marine areas^7–11^ illustrating that the link between trophic conditions and ecology of AAP bacteria is not trivial. Indeed, besides the unifying property to perform light-dependent energy transduction, AAP bacteria are very diverse in terms of physiology and metabolism^2,12^.

To gain a more accurate picture of the factors governing the ecology of AAP bacteria, their diversity was investigated in different oceanic regions (*e.g*. refs^10,13–16^), and several studies have attempted to connect the observed patterns with environmental variables (*e.g*. refs^10,15^). Although some trends began to emerge, the link between ecological niches, phylogeny and habitat of AAP bacteria remains patchy. Most studies hypothesized that multifactorial parameters, such as geographic distance (*e.g*. refs^16,17^) and environmental gradients (*e.g*. refs^15,18–20^) act in structuring AAP populations, but the respective importance of these factors has not really been explored. Moreover, recent reports suggest that the expected diversity of AAP bacteria was overestimated and that their biogeographical patterns are not so obvious^15,20,21^.

Determining how environmental conditions control the ecology of AAP bacteria, particularly at a fine taxonomic resolution (*i.e*., sufficient to identify lineages with distinct traits), is critical for understanding how these organisms populate the oceans and contribute to global carbon cycling. Following this idea, the objectives of the present study were (i) to provide elements establishing the role of the environmental context in structuring AAP bacterial diversity, (ii) to prioritize the processes (*i.e*, deterministic *vs* stochastic) responsible for generating AAP bacterial patterns in marine environments, (iii) to determine if a link between phylogeny and habitat preferendum exists. To this aim, we analyzed the sequence polymorphism of the gene encoding for the *M* subunit of the core photosynthetic apparatus (*pufM* gene) of AAP bacteria in contrasted oceanic provinces encompassing different marine regions, distinct nutrient status and oceanic regimes, various temperatures, salinities and depths.

## Results

**Figure 1:**
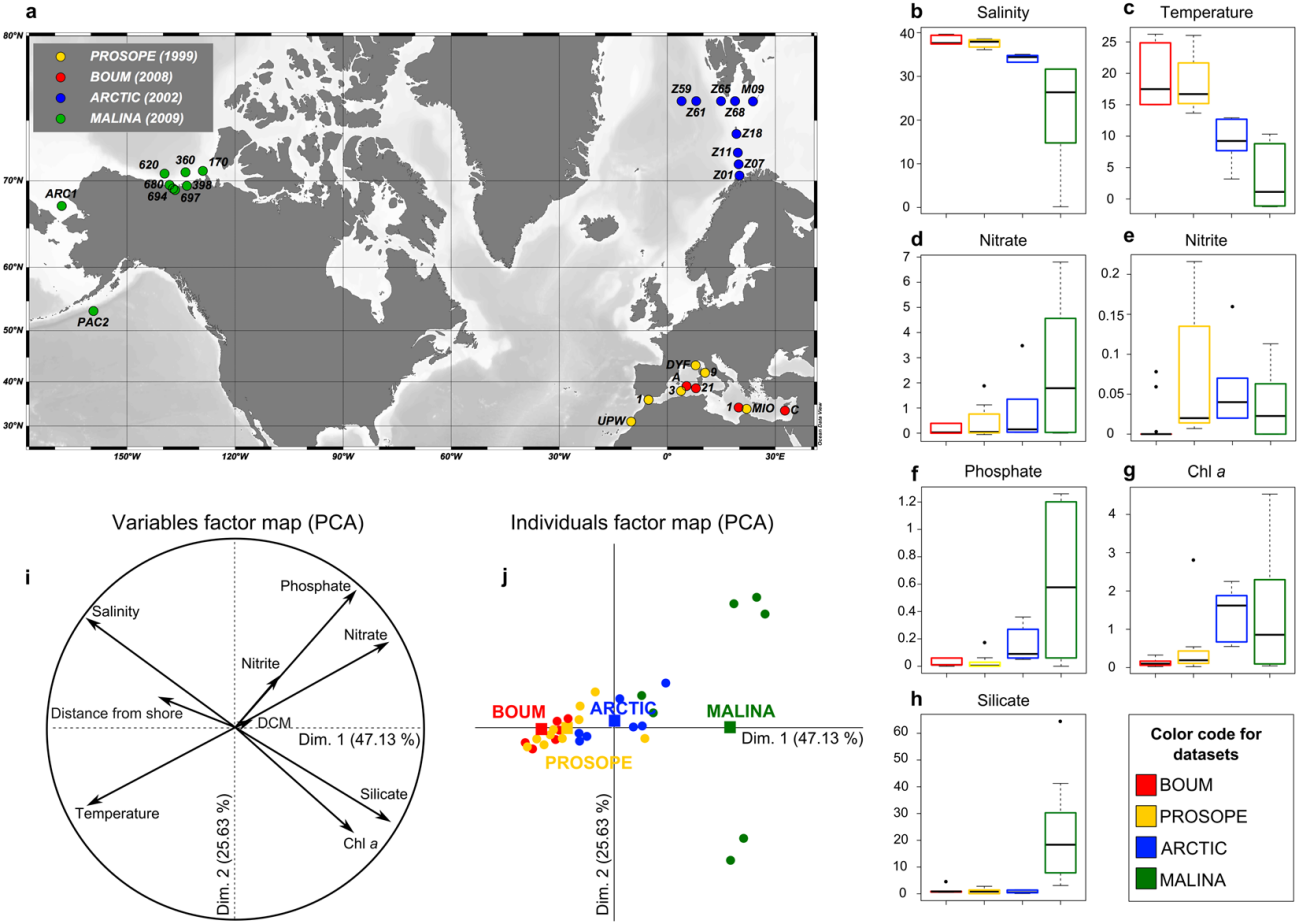
(**a**) Schematic representation of the location of stations sampled during cruise tracks depicted on Ocean Data View (http://odv.awi.de)^45^, map. (**b**–**h**) Box-plots displaying for each dataset: (**b**) Salinity (in g.L^−1^), (**c**) Temperature (in °C) and concentrations (in µmol.L^−1^) in (**d**) Nitrate, (**e**) Nitrite, (**f**) Phosphate, (**g**) Chlorophyll *a* (Chl *a*) and (**h**) Silicate. (**i**) Variables factor map and (**j**) Individuals factor map of the principal component analysis (PCA) performed using standardized quantitative values of the following quantitative ancillary variables: temperature (in °C), salinity (g.L^−1^), chlorophyll *a* (in µM), silicate (in µM), nitrite (in µM), nitrate (in µM) and phosphate (in µM); qualitative data (depth of Deep Chlorophyll Maximum (DCM) and distance to shore) were used as illustrative factors.

The *pufM* dataset used in this study was assembled from sequences previously generated from 27 samples collected during 4 cruises (PROSOPE, BOUM, ARCTIC and MALINA) which took place between 1999 and 2009 in different oceanic regions (Mediterranean, North Pacific to Western Beaufort Sea, Barents and Norwegian seas; Fig. 1a). The cruise transects encompass three major oceanic biomes (polar, westerly winds and coastal boundary zone) and six oceanic provinces [Atlantic Subarctic, Atlantic Arctic, Boreal Polar, Canary coastal, Pacific Subarctic gyres and Mediterranean Sea-Black Sea, Table S1] encompassing large ranges of salinities (Fig. 1b) and temperatures (Fig. 1c), distinct nutrient status (Fig. 1d–h) and oceanic regimes (Table S1) and various depths (Table S1).

All of the ~300 *pufM* sequences were generated by classical cloning-sequencing approaches after amplification by a same primer pair, to give 245 bp PCR products, avoiding biases in the comparison of the relative abundance of OTUs. The sequences were grouped into OTUs, at 94% nucleic acid sequence similarity, using the furthest neighbor clustering method. The number of OTUs in each set of *pufM* sequences was variable (20 to 48 OTUs), presumably due to a different number of sequences in the primary datasets (Table S2). Despite those differences, coverage values indicated that most diversity in most sequence sets has been retrieved (Table S2). A total of 107 OTUs were identified for the overall dataset with a coverage value of 96% (Table S2, Supplemental material SI1). Only 6 OTUs exhibited a degree of paraphyly (paraphyly index (PI) comprised between 0.01 and 0.08), the other 101 OTUs were monophyletic (PI = 0, data not shown).

### Comparisons of *pufM* datasets

**Figure 2:**
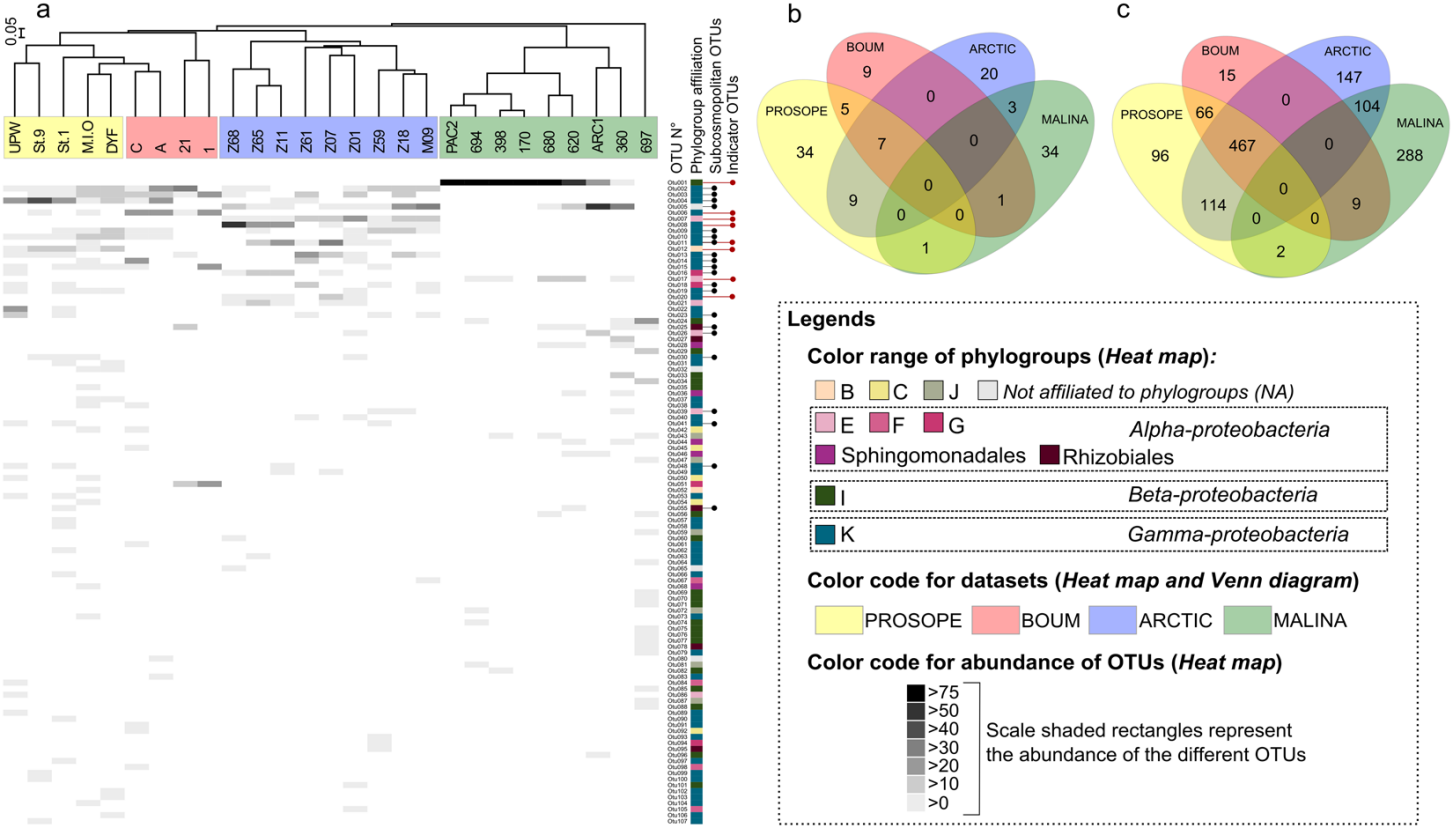
(**a**) Hierarchical clustering (Bray-Curtis distance) of stations according to OTU relative abundance and heat map showing OTU abundance and distribution. Subcosmopolitan OTUs (OTUs found in at least 2 oceanic regions) and indicator OTUs [according to the concept of indicative species (Auguet *et al*. 2010)] are indicated at the right. (**b**–**c**) Venn diagrams showing the numbers (**a**) of unique and shared OTUs and (**b**) of unique and shared *pufM* sequences between PROSOPE, BOUM, ARCTIC and MALINA datasets, respectively.

We used community ecology methods to analyze the structure of AAP bacterial communities using two metrics that each emphasized different community characteristics. Bray-Curtis provides a measure of community composition differences between samples based on OTU counts, regardless of taxonomic assignment^22^. UniFrac quantifies community similarity based on the phylogenetic relatedness^23^. Both methods showed that AAP bacterial communities exhibited divergent structure of their diversity (Fig. 2a) and phylogenetic composition (Fig. S1) over stations. Nevertheless, at a broader scale, each oceanic region consistently clustered together (Fig. 2a, Fig. S1) illustrating that AAP bacterial signatures were unique to each oceanic region (Mediterranean Sea, Beaufort, Barents and Norwegian seas) (Fig. 2a).

To obtain deeper insights into the differences in AAP bacterial community composition, we also assessed the number of shared OTUs and sequences between marine regions. No OTU was common to all oceanic areas investigated here and many were only detected in one sample (Fig. 2a,b). A few dominant OTUs were shared across oceanic regions (Fig. 2b,c). For example, the 7 OTUs common to the Mediterranean (PROSOPE and BOUM datasets) and the Barents and the Norwegian Seas (ARCTIC dataset) covered more than 56% of the *pufM* sequences retrieved from these oceanic biomes (Fig. 2b,c). Similarly, the 3 OTUs shared by the ARCTIC and MALINA datasets grouped 104 *pufM* sequences (~20% of sequences; Fig. 2b,c). OTUs common to at least two oceanic regions, hereafter identified as subcosmopolitan OTUs, were affiliated to *α-* and *γ-Proteobacteria* within similar proportions (25% and 28%, respectively, Fig. 2a). In contrast, all OTUs affiliated to *β-Proteobacteria* were detected in only one oceanic region (Fig. 2a). Applying the ecological concept of indicator species according to Auguet *et al*.^24^ (*i.e*. specialist lineages the most frequently represented in most sites of an oceanic region), we identified only 8 indicator lineages (significant IndVal index (P < 0.05)) for the four marine environment analyzed (Fig. 2a). Half of them (OTUs 007, 008, 011, 020) were characteristic of the Barents and Norwegian seas, 2 (OTUs 001, 017) of the western Beaufort Sea, and the OTUs 006, 012 were specific of the Mediterranean Sea (Fig. 2a).

### Spatial species turnover

**Figure 3:**
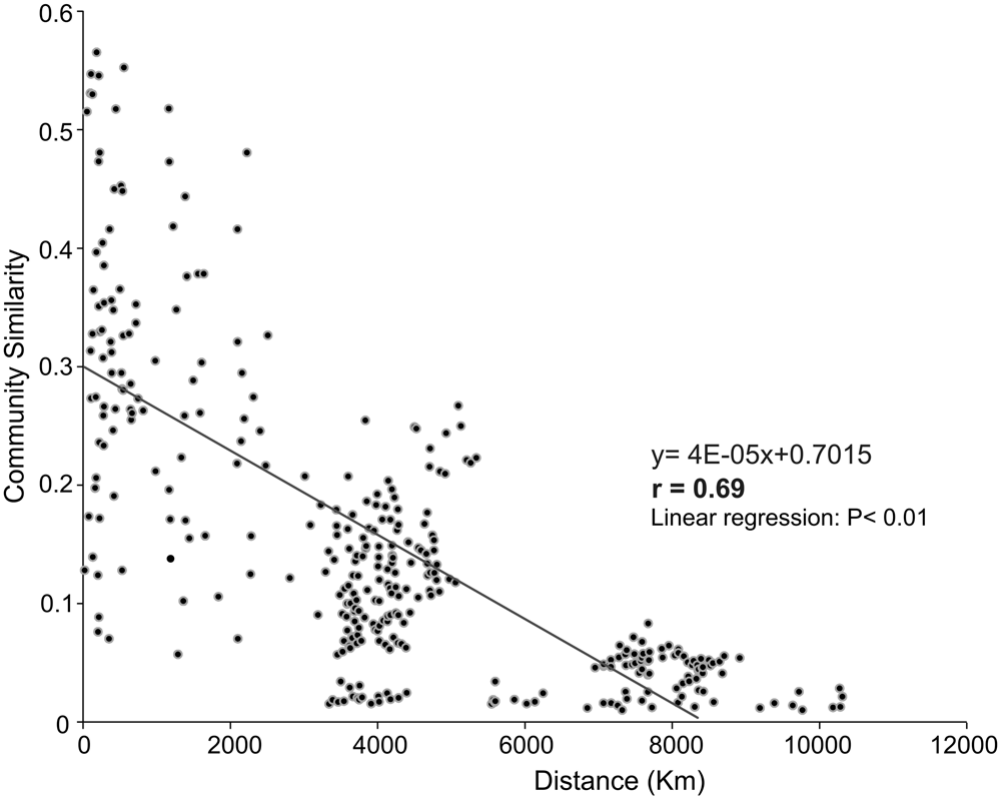
Distance-decay curves for AAP bacterial communities. The solid black line denotes the least-squares linear regression across all spatial scales. Linear regression: P < 0.01.

The AAP community similarity between each pairwise set of samples decayed significantly (p < 0.000001) with the geographic distance (Fig. 3). To deeply understand the link between AAP bacterial communities and geographical distance, we compared them using similarity matrices and Mantel tests. Distance matrices for environmental variables and geographic distance were measured by the Euclidean distance between values at two stations. We used Mantel tests to determine the correlation between species similarity matrices and environmental and geographic distance (Table 1). The Mantel correlation (Mc) between species similarity and geographic distance (0.75, p = 0.000001) was higher than with environmental factors (0.45, p = 0.000001). Simple Mantel tests also revealed that environmental factors and geographic distance were significantly correlated (0.38, p = 0.000001). However, the Mc between species similarity and geographic distance, partialling out environmental factors, was not significant (0.08, p = 0.1). In contrast, Mc between environmental factors and species similarity, independent of distance, was significant (0.25, p = 0.00006) (Table 1).

**Table 1:** Mantel and partial Mantel tests between species similarity and environmental determinants and geographical distance.

	**Simple Mantel tests**				**Partial Mantel tests**			
	Environmental factors		Geographic distance		Environmental factors		Geographic distance	
	*Mantel r*	*P-value*	*Mantel r*	*P-value*	*Mantel r*	*P-value*	*Mantel r*	*P-value*
Species similarity	0.45	0.000001	0.75	0.000001	0.25	0.00006	0.08	0.1
Environmental factors	—	—	0.38	0.000001	—	—	0.70	0.00001

### Environmental gradients driving AAP bacteria structure and identification of AAP bacteria ecoclades

**Figure 4:**
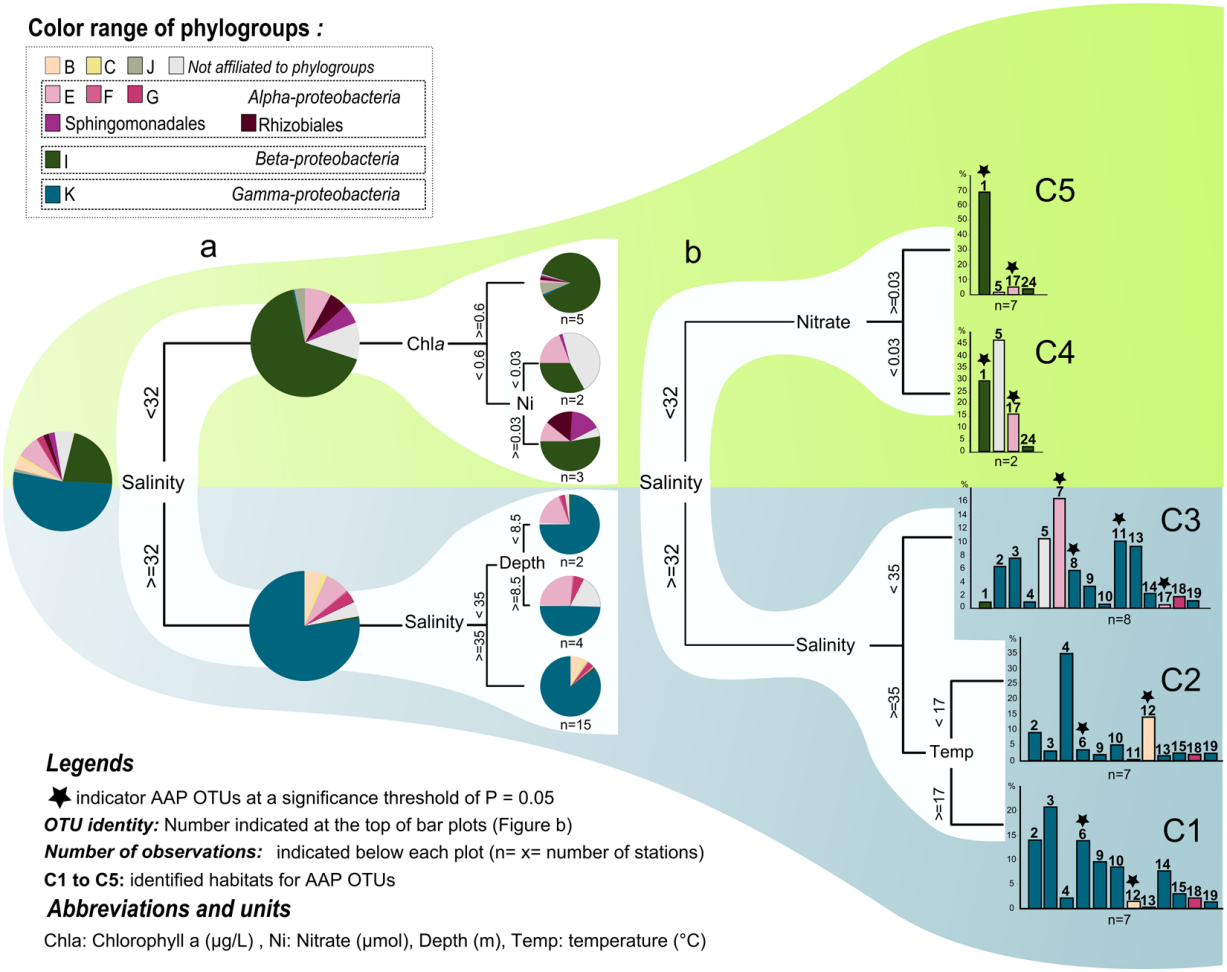
Multivariate regression tree (MRT) analyses of the interaction between environmental parameters and (**a**) AAP bacterial phylogroups and (**b**) OTUs abundance (in terms of sequence number). The model explained 89% and 61% of the variance in the whole data set for phylogroups and OTUs, respectively. Pies (**a**) and multiple value bar chars (**b**) under each leaf represent the mean of normalized phylogroups (**a**) and OTUs (**b**) abundance for each lineage significantly correlated with environmental parameters. Number on the bar chars (**b**) indicated the number of the corresponding OTUs. Asterisks show indicator OTUs at a significance threshold of P = 0.05. C1 to C5 indicated the 5 habitats identified for AAP from the MRT analysis (*e.g*. habitat C1: salinity ≥35 g.L^−1^ and temperature ≥17 °C). [*Note that stations Z65 and Z59 from ARCTIC and stations ARC2 and PAC2 from MALINA were not used in MRT analyses because of the lack of environmental data (see* Table S1)].

Multivariate regression trees (MRT) were performed to explore and predict relationships between the relative abundance of phylogroups (Fig. 4a) and of OTUs (Fig. 4b) to environmental determinants. The MRT analysis for phylogroups showed a six-leaf tree ordination (explaining 89% of the standardized variance) primarily based on salinity, and followed by Chl*a*, nitrate and depth (Fig. 4a). Pie charts show how the relative abundance of each phylogroup contributed to the separation and composition of the leaves (Fig. 4a). The MRT analysis carried out for OTUs explained 61% of the standardized variance, with salinity, temperature and nitrate mainly responsible of branch splits (Fig. 4b). Whether for phylogroups or OTUs, salinity explained a significant part of the distribution patterns (71% for phylogroups, 32% for OTUs, Fig. 4). The AAP bacteria belonging to γ- and *β-Proteobacteria* roughly shared more similar distribution patterns within the lineage than between lineages whereas members of *α-*AAP were widespread (Fig. 4). The indicator OTUs (Fig. 2a) were mainly responsible for the regression tree topology observed and enabled us to identify 5 habitat categories (C1 to C5, Fig. 4b, Supplemental material SI1).

**Figure 5:**
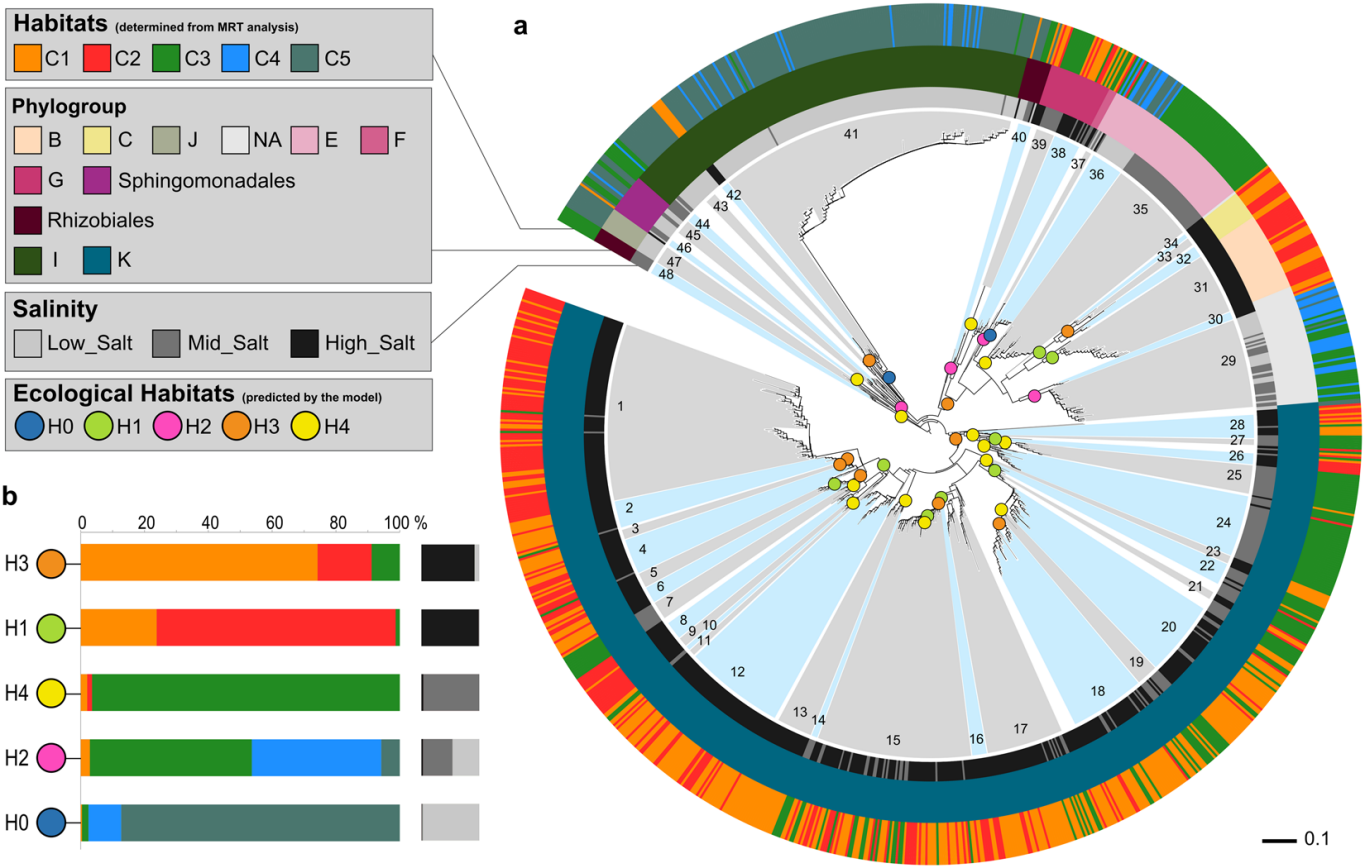
(**a**) Maximum likelihood phylogenetic tree showing environmental characteristics and habitat predictions. Characteristics of the sampling locations are plotted on the inner and outer rings: the inner ring indicates the phylogroup, the intermediate ring indicates high (≥35 ppt), medium (32≤ salt ≤35) or low (<32 ppt) salinity and the outer ring indicates the microenvironment (according to the identified habitat C1 to C5 in the MRT analysis). Ecological habitats predicted by the model are identified by colored circles at the parent nodes on the tree. Ecoclades which contained at least 10 *pufM* sequences and passed a post hoc empirical significance threshold (*P-value* < 0.01) are indicated by alternating blue and gray shading of clusters. (**b**) The distribution of each habitat among microenvironments and salinity. The habitat and environment colors match the legend in (**a**).

In MRT analyses, most of the variance of the AAP bacterial composition was explained, at different taxonomic level, by environmental parameters (Fig. 4). This suggests that it should be possible to demarcate *pufM* sequences into ecologically cohesive clades, sharing a common projected habitat which reflected their relative abundance in the environmental categories defined previously (C1 to C5, Fig. 4b, Supplemental material SI1). We used AdaptML to demarcate ecoclades by inferring the evolutionary history of habitat transitions. The resulting observations suggest that AAP bacteria resolved into a striking number of ecologically distinct ecoclades with clearly identifiable preferences (Fig. 5a). The analysis identified 5 distinct inferred habitats (H0 to H5), for the 48 ecoclades, with strong signals from salinity and other environmental settings (Fig. 5b). The OTUs affiliated to *α-* and *β-like* AAP bacteria did not contain mixed environmental signal and ecoclades represented coherent phylogenetic clusters. However, numerous dominant γ-like AAP bacterial populations exhibited distinct habitat preferences within a same OTU (Figs 5a, S2).

## Discussion

Recent results gathered from sampling expeditions (*e.g*. refs^17,18,20^) and from high-throughput sequencing approaches to achieve *pufM* gene polymorphism (*e.g*. refs^17,19,25^) question the actual extent of the diversity but also the existence of biogeographical patterns for AAP bacteria in marine systems. Indeed, (i) most *pufM* sequences identified from newly explored ecosystems, including some isolated and extreme environments, are similar to sequences retrieved elsewhere (*e.g*. refs^17,18,20^), (ii) next generation sequencing approaches such as 454 pyrosequencing^19,25^ or Illumina MiSeq amplicon^17^ did not yield a significant higher number of OTUs than classical clone library methods (*e.g*. refs^15,16,26^). We previously addressed these issues and highlighted the need to rely on comprehensive analyses integrating comparable datasets of *pufM* sequences from different oceanic regions^20^. This study is in this line and although the sampling was not exhaustive, it offers a comparison of the diversity of AAP bacterial communities in different oceanic provinces and it allows us to identify factors shaping their structure across different marine biomes in the northern hemisphere.

We observed that AAP bacteria exhibited a significant turnover of taxa (*beta-diversity*) across the oceanic regions investigated here providing strong evidence of biogeographical patterns for these microbes, the distance-decay relationship being one example (Fig. 3). Selection and dispersal of microbes are commonly accepted as main drivers of biogeographical patterns^27^. To further investigate the underlying mechanisms of AAP bacterial patterns, we tested whether samples were more similar within than across ocean regions. If the dispersal limitation rather than the environmental selection dominated, a higher similarity within than across ocean regions was expected. By contrast, if the environmental selection fully explained biogeographic patterns, we would expect environmental factors to correlate with community similarity.

Our analyses revealed that the environmental selection was the largest main-effect factor contributing to AAP bacteria taxonomic variation between oceanic regions (Table 1). However, the interaction of environment and distance better explained the variation than either of the main-effect factors, indicating a role for as yet unexplained covariance between environment and separation distance (Table 1). We also identified subcosmopolite OTUs (*i.e*., OTUs shared by at least two oceanic regions) which were mainly representative of dominant AAP bacterial populations (Fig. 2). In line with Bibiloni-Isaksson *et al*.^17^, this result could reinforce the idea that key AAP bacterial groups are widely distributed across similar environments.

Overall, our results suggest that geographic distance plays a subordinate role on the composition of AAP marine microbial communities and rather selective processes shape their community composition, a hypothesis summed up by microbiologists as, “*everything is everywhere-the environment selects*”^28,29^. Therefore, a distance-decay curve is observed because environmental variables tend to be spatially auto correlated and AAP bacteria with differing niche preferences are selected from the available pool of taxa as the environment changes with distance.

Accordingly, deterministic processes (*i.e*., selection by environmental variables) are important for non-random spatial distributions of marine AAP bacteria, suggesting that these microbes are specialized on particular habitats. This is consistent with former meta-analyses on natural bacterial assemblages (*e.g*., refs^30–32^). We showed well-defined community patterns along broad environmental conditions and habitat types (Fig. 4). Taken together, our data revealed salinity as the major environmental factor shaping taxonomic AAP bacterial community composition in the ocean (Fig. 4) while trophic status (*i.e*., Chl *a* and nitrate concentrations) and temperature were subsequent explaining factors (Fig. 4). Therefore, our analysis highlighted that physiological constraints play a key role in the AAP bacterial assemblages, and beyond, salinity especially seems to encourage such lineage-specific adaptations. Previous studies on prokaryotic assemblages have shown that salinity is the major determinant structuring bacterial^30^ and archaeal communities^24^. Its influence exceeded that of temperature and/or light, recognized as selective parameters for some of other marine microbial taxa^33,34^. Such commonality in the types of determinant factors suggests that, at global scale, adaptative strategies (physiological constraints) determine the occurrence of AAP in accordance to their heterotrophic status. This does not exclude that at a local scale, other determinants (*e.g*., light) act in structuring AAP bacterial populations (*e.g*., refs^15,18,20,21^).

The AAP bacteria belonging to γ- and *β-proteobacteria* roughly shared more similar distribution patterns (and probably more similar physiological traits) within the lineage than between lineages whereas members of *α-*AAP bacteria were widespread. For example, γ- and *β-*AAP bacteria favor high and low level of salinity, respectively. The preference of *Betaproteobacteria* for low salt levels is consistent with previous studies that reported their dominance in brackish and freshwater environments [3] and references therein). But by highlighting the predilection of gammaproteobacterial AAP bacteria for high salinity marine systems, this analysis sheds a new light on their ecology. This finding is well supported by their overwhelming dominance in the Mediterranean Sea^15,35^ where salinity ranged from 36.2 to >39‰^36^ and provides an interesting framework for designing future culture efforts to expand the diversity of cultivable γ-AAP bacteria.

A global dispersal potential for microorganisms^37^ and subsequent environmental selection may represent a mechanism for driving patterns of microbial biogeography^34^. At the same time, local adaptations by natural selection will lead to differences in spatially distant populations of phylogenetically similar organisms^34^. We found that AAP bacteria resolve into a striking number of ecologically distinct ecoclades with clearly identifiable preferences (Fig. 5a). We identified five distinct inferred habitats (H0 to H5) with strong signals from salinity and other environmental settings (Fig. 5b). OTUs affiliated to *α-* and *β-*like AAP bacteria did not contain mixed environmental signal and ecoclades are coherent in terms of phylogeny indicating that ecological niches for AAP bacteria are expressed at the species (*i.e*., OTU) level. However, numerous dominant γ-like AAP bacterial populations exhibited distinct habitat preferences within a same OTU, reinforcing the previously suggested idea^15^ that ecotypes exist for γ- AAP bacteria.

It is quite surprising to find both phylogenetic and ecological coherence within AAP bacterial populations, since those affiliated to *α-* and *β-*Proteobacteria, expressing a photosynthetic gene cluster supposed to be submitted to lateral gene transfers (LGT). The LGT theory was actually pointed out to be a reasonable and likely hypothesis to explain the patchy distribution of photosynthesis among different bacterial lineages^38^. But, our ecological interpretation favors the hypothesis of recurrent losses of photosynthetic capacity in different lineages, descendant from a photosynthetic common ancestor^39^. Indeed, the congruence between habitat and evolutionary relatedness suggest that AAP bacteria acquired their phototrophic abilities a long time ago. That scheme also enables us to better understand why AAP are phototrophs, as the ecological benefit of their phototrophy is not clear yet. This hypothesis is consistent with recent analyses suggesting that the capacity to synthesize Bchl *a* originated only once in a phototrophic bacterium that pre-dated -at the very least- the radiation event that gave rise to the phylum Chloroflexi, Chlorobi, Acidobacteria, and Proteobacteria^40^. This places Bchl *a* synthesis at an early stage during the evolution of bacteria and implies that phototrophy might have been a common trait in ancestral populations of bacteria during the Archean^40^. However, γ-like AAP challenge that hypothesis (Fig. 5). This result suggests that gammaproteobacterial AAPs acquired phototrophic capacity via a different evolutionary scenario and may evolve under different constraints illustrating again that the evolution of phototrophy in Proteobacteria is a very complex process^3,41–43^. To explore this question, comparing whole genomes of gammaproteobacterial AAPs will be important to identify specific changes leading to adaptative evolution.

The identification of ecoclades is a major advance in the understanding of the ecology of AAP bacteria. Since our analysis was based on a dataset of only 1,306 sequences and four marine regions, we acknowledge that ecoclade identification should be controversial. To determine if these ecoclades have an ecological consistency beyond the oceanic regions investigated here, a set of *puf*M sequences amplified from surface waters of the Pacific Ocean^16^ was added to the primary dataset. Although these *puf*M sequences were not obtained using the primer set that we used, 76% of them fell in 9 predicted ecoclades (ecoclade No. 1, 11, 12, 14, 17, 27, 35, 38, and 47). These ecoclades are mainly affiliated to *Gammaproteobacteria* with projected habitats H0 (15%), H3 (46%) and H4 (15%). The moderate to high salinity levels of these projected habitats are consistent with the environmental settings reported by the authors^16^.

In conclusion, our results clearly indicate a dominant role of deterministic processes in influencing the continental-scale structuring of AAP bacteria at different taxonomic levels and reveal that AAP bacteria show strong habitat associations that have likely emerged through evolutionary adaptation. Moreover, we showed that the distribution and structure of AAP bacterial communities can largely be understood in terms of habitat properties solely allowing identifying cohesive ecological clades with a surprising ecological and phylogenetic coherence. This result suggests that it would be possible to predict AAP bacterial assemblages from habitat properties in the marine environment.

## Methods

### Brief description of the pufM dataset

The dataset was assembled from studies^15,18,20,35^ examining AAP bacterial communities using *puf*M sequences amplified using the *PufMF* forward^13^ coupled with the *PufM_WAW* reverse^44^ primers to give a ~245 bp PCR product. For more details on PCR amplification conditions and clone library construction, see Lehours *et al*.^15^. We analyzed a total of 1,306 *pufM* sequences (see supplemental informations) for which at least one representative sequences of each operational taxonomic unit (OTU) are available in Genbank under accession n° HQ871842-HQ871863, JF421730-JF421749, GQ468944-GQ468986, JN248465-JN248539, and KM654564-KM654598.

### Sampling locations and ancillary data

The *pufM* sequences were recovered from samples collected during four oceanographic cruises, namely PROSOPE^15^ and BOUM^35^ in the Mediterranean Sea, MALINA^18^ from the North Pacific Ocean to the Western Beaufort Sea, and ARCTIC^20^ at the boundary between the Norwegian, Greenland, and Barents Seas. The location of the stations sampled during the four cruise tracks are depicted on the Ocean Data View (http://odv.awi.de)^45^, map (Fig. 1a). Stations were affiliated to oceanic biomes and to oceanic provinces defined by Longhurst^46,47^ using the Longhurst Biogeographical map of arcgis (http://www.arcgis.com). The geographical distances between stations were calculated using the Geographic Distance Matrix Generator (http://biodiversityinformatics.amnh.org/open_source/gdmg/download.php). More details on the area sampled, the sampling procedures and the ancillary parameters characterizing each sampling area were described previously^15,18,20,35^.

The main ancillary parameters characterizing each sampling area, summarized in Table S1, were plotted in box plots generated using R software^48^ and used to perform a Principal Component Analysis (PCA) generated using FactoMineR package (http://cran.r-project.org/web/packages/FactoMineR/index.html).

#### Sequence clustering into OTU

A conservative value of 94% nucleic acid sequence similarity^49^ was chosen for clustering the 1,306 *pufM* sequences into Operational Taxonomic Units (OTUs) using the furthest neighbor clustering method. Sequence clustering was performed using MOTHUR (http://www.mothur.org/)^50^. MOTHUR was also used to generate a heatmap displaying the relative abundance of each OTU for each station. To compensate for the sequencing depth bias per sample in the heatmap generation, sequence abundance values within each OTU were normalized for comparison of OTU abundance between samples. A clustering, based on OTU composition and abundance (Bray-Curtis distance) at the different stations, was also performed and plotted on top of the heatmap. The Venn diagram was generated using MOTHUR and an “in house” developed Perl script.

#### Phylogenetic analyses of *pufM* sequences

All *pufM* sequences were aligned using ARB (http://www.arb-home.de/)^51^, and added using ADD-BY-PARSIMONY algorithm to a robust *pufM* tree constructed as described previously^15^. Phylogenetic tree display and annotation were performed with iTOL software (http://itol.embl.de/)^52^. AAP bacterial communities retrieved from the 4 studies (PROSOPE, BOUM, ARCTIC and MALINA) were compared using phylogenetic information with Unifrac distance metric (http://bmf2.colorado.edu/unifrac/index.psp)^23^. The following phylogenetic analyses were performed with Unifrac^1^: “Unifrac significance”, comparing each pair of environments, which gives a P-value dissimilarity matrix^2^, “Cluster environments” determining using UPGMA method which environments have similar microbial communities^3^, “Jackknife environment clusters” performing Jackknife analysis of environment clusters (100 resamplings) produced with the Cluster environments analysis option to determine the robustness of the analysis. We calculated the paraphyly index (PI) as described by Koeppel & Wu^53^.

#### Resemblance matrices for biogeographic analyses

Three square resemblance matrices were performed using R software^48^: the biotic similarity matrix (derived from OTU abundance using Morista-Horn index), the environmental-similarity matrix (derived from environmental data matrix after standard normalization and using Euclidean distance), the geographic distance matrix (derived from the site-location matrix including latitude and longitude values for each station). The environmental parameters integrated were the following: depth, salinity, concentrations in nitrate, phosphate, nitrite, silicate and chlorophyll *a*, distance to shore, and the position according to DCM (Table S1). To investigate the relationship between AAP bacterial community similarity, geographic distance, and environmental distance across spatial scales, we performed simple and partial Mantel tests with 1,000,000 iterations, using zt software (http://bioinformatics.psb.ugent.be/software/details/ZT)^54^.

#### Taxon-based approach and demarcation of “ecoclades”

To identify AAP bacterial taxa which may be analogous to the concept of ‘indicator species’^24^, tables of relative abundances for phylogroups and for OTUs were constructed. We used the indicator value (IndVal) index, which combines the relative abundance and relative frequency of occurrence of OTUs^55^. Multivariate regression trees were computed with the R package mvpart^56^ in order to represent the relationship between phylogroup or OTU relative abundances and the environmental matrix. The quantitative environmental parameters used in the MRT were the following: depth, salinity, concentrations in nitrate, phosphate, nitrite, silicate and chlorophyll *a*, latitude and longitude. The qualitative parameters used in the MRT were: the trophic status of the sampling location (defined according to phosphate concentrations as eutrophic (>20 µg.L^−1^), mesotrophic (10–20 µg.L^−1^) or oligotrophic (<10 µg.L^−1^)); the distance to shore (coastal or offshore), the oceanic region and the sampling depth relative to the DCM (Table S1). We used AdaptML (available at http://almlab.mit.edu/adaptml/)^57^, to demarcate ecoclades in our marine AAP bacteria dataset. AdaptML is a maximum likelihood method that employs a hidden Markov model to learn ‘projected habitats’ (distribution patterns among environmental categories) and ecologically cohesive ‘populations’ (groups of related strains sharing the same projected habitat). Our AdaptML analysis used the 5 habitats (C1 to C5) predicted from the MRT analysis. The habitat learning and clustering steps of AdaptML were performed using the default settings.

### Data availability

Sequence representatives of each OTU are available in Genbank under accession n° HQ871842-HQ871863, JF421730-JF421749, GQ468944-GQ468986, JN248465-JN248539, and KM654564-KM654598. All sequences, ADAPTML results and OTU cluster file are provided as supplemental material (Supplemental material SI1, Fig. S2)

## Electronic supplementary material

Supplemental Material

Dataset 1
