# Supplementary material for: Biogeographic patterns of aerobic anoxygenic phototrophic bacteria reveal an ecological consistency of phylogenetic clades in different oceanic biomes
Source: Sci Rep. 2018 Mar 7;8:4105. doi: 10.1038/s41598-018-22413-7 (PMC5841314; doi:10.1038/s41598-018-22413-7)
Supplement: Supplementary file 1 — Supplemental Material [file 41598_2018_22413_MOESM1_ESM.pdf]

SUPPLEMENTAL MATERIAL

Biogeographic patterns of aerobic anoxygenic phototrophic bacteria reveal an ecological consistency of phylogenetic clades in different oceanic biomes

Lehours Anne-Catherine, Enault François, Boeuf Dominique, Jeanthon Christian

Table S.1. Some features of the sampling stations. DCM: Deep Chlorophyll Maximum, NA: not determined, OLIGO: Oligotrophic status, MESO: Mesotrophic status, EUT: Eutrophic status. Latitude and longitude are expressed in decimal degrees. Oceanic biomes and oceanic provinces were defined according to Longhurst (2007): Oceanic biomes: polar, westerly winds (westerlies) and coastal boundary (coastal) domains. Oceanic provinces: SARC = Atlantic sub-Arctic; ARCT = Atlantic Arctic; BPRL = Boreal polar; MEDI = Mediterranean Sea; PSAE = Eastern Pacific subarctic gyres; CNRY = Canary current coast.

Table S.2. Properties of the distribution of *pufM* sequences at each sampling station. Coverage was calculated as follows: Cx = 1-(nx/N) where nx is the number of clone types that are encountered only once in library x and N is the total number of clone analyzed.

Figure S.1: Hierarchical clustering of samples based on phylogenetic similarity between AAP bacterial communities and dissimilatory matrix showing the significance of *p*-values**.** Values on nodes represent confidence values of clusters (Jackknife counts, 100 resamplings). [Note that stations St.1 and St.21 from BOUM dataset were excluded from the analysis because of their low number of sequences (see Table S2)].

Figure S.2: Regular view of the maximum likelihood phylogenetic tree presented Figure 5 (Legends and colours are similar to Figure 5). The ADAPTML_N° of sequences (see Supplemental information SI1) are indicated.

Dataset	Station	Oceanic biome	Oceanic province code	Depth (in m)	Nitrite (in µM)	Nitrate (in µM)	Phosphate (in µM)	Silicate (in µM)	Temperature (in °C)	Salinity (in g.L-1)	Chl a (in µM)	Position	Distance to shore (in Km)	Trophic status	Latitude	Longitude
ARCTIC	Z01	Polar	SARC	5	0.020	0.040	0.060	0.390	12.92	33.19	0.61	aboveDCM	23	OLIGO	70.5007	20.0218
	Z01	Polar	SARC	12	0.040	0.150	0.090	0.520	12.70	33.25	0.76	aboveDCM	23	OLIGO	70.5007	20.0218
	Z11	Polar	SARC	25	0.160	3.500	0.360	1.380	9.23	34.79	1.82	inDCM	225	EUT	72.502	19.5728
	Z18	Polar	ARCT	35	0.040	0.760	0.270	1.550	3.18	34.41	0.55	belowDCM	38	EUT	73.9992	19.2097
	M09	Polar	BPLR	20	0.070	0.980	0.200	1.280	4.63	34.47	2.23	inDCM	106	MESO	76.3095	23.783
	Z68	Polar	ARCT	15	NA	NA	NA	NA	7.69	34.24	1.88	belowDCM	54	NA	76.3333	18.7833
	Z65	Polar	ARCT	25	NA	NA	NA	NA	8.17	34.84	1.62	belowDCM	41	NA	76.3333	14.8833
	Z61	Polar	ARCT	5	0.050	1.400	0.260	0.900	8.24	35.04	2.25	aboveDCM	180	EUT	76.3327	7.9963
	Z59	Polar	ARCT	35	NA	NA	NA	NA	3.59	34.67	2.24	aboveDCM	280	NA	76.3317	3.9866
	Z07	Polar	SARC	5	0.020	0.020	0.050	0.070	11.74	34.42	0.67	aboveDCM	132	OLIGO	71.501	19.8025
BOUM	St.1	Westerlies	MEDI	50	0.000	0.000	0.000	4.770	16.50	38.17	0.10	aboveDCM	210	NA	34.3277	19.82
	St.21	Westerlies	MEDI	3	0.002	0.028	0.028	0.890	24.86	37.34	0.06	aboveDCM	63	OLIGO	38.6308	7.9097
	St.21	Westerlies	MEDI	85	0.079	0.130	0.032	0.640	14.96	37.80	0.33	inDCM	63	OLIGO	38.6308	7.9097
	St.A	Westerlies	MEDI	3	0.000	0.010	0.010	0.930	24.35	37.42	0.06	aboveDCM	177	OLIGO	39.0993	5.35
	St.A	Westerlies	MEDI	90	0.000	0.390	0.010	0.640	15.04	37.62	0.17	inDCM	177	OLIGO	39.0993	5.35
	St.C	Westerlies	MEDI	3	0.000	0.000	0.060	0.860	26.23	39.65	0.03	aboveDCM	114	OLIGO	33.625	32.6533
	St.C	Westerlies	MEDI	100	0.060	0.140	0.060	0.550	17.49	39.40	0.3111	inDCM	114	OLIGO	33.625	32.6533
MALINA	ARC1	Polar	BPLR	0	NA	NA	NA	NA	NA	NA	NA	aboveDCM	112	NA	67.49	-168.12
	PAC2	Westerlies	PSAE	0	NA	NA	NA	NA	NA	NA	NA	aboveDCM	250	NA	53.36	-159.29
	St.170	Polar	BPLR	5	0.000	0.037	0.660	9.405	3.50	29.21	1.05	aboveDCM	110	EUT	70.91	-128.92
	St.360	Polar	BPLR	3	0.000	0.020	0.577	3.094	-0.10	26.36	0.05	aboveDCM	125	EUT	70.8032	-133.7323
	St.360	Polar	BPLR	60	0.113	4.600	1.210	12.452	-1.12	31.74	0.28	inDCM	125	EUT	70.8032	-133.7323
	St.398	Polar	BPLR	0	0.011	0.700	0.111	30.273	8.80	16.37	4.53	aboveDCM	12	MESO	69.5142	-133.4185
	St.620	Polar	BPLR	3	0.000	0.000	0.330	7.821	1.13	22.09	0.09	aboveDCM	120	EUT	70.6814	-139.6215
	St.620	Polar	BPLR	80	0.026	6.800	1.260	18.340	-1.14	31.67	0.04	belowDCM	120	EUT	70.6814	-139.6215
	St.680	Polar	BPLR	3	0.000	0.030	0.000	3.517	8.04	14.77	0.18	aboveDCM	27	OLIGO	69.6059	-138.2349
	St.680	Polar	BPLR	45	0.063	6.330	1.201	18.763	-1.25	31.66	0.86	aboveDCM	27	EUT	69.6059	-138.2349
	St.694	Polar	BPLR	0	0.072	1.780	0.061	41.267	9.28	9.43	2.30	aboveDCM	30	OLIGO	69.25	-137.1959
	St.697	Polar	BPLR	0	0.023	3.600	0.020	64.525	10.32	0.15	2.95	aboveDCM	17	OLIGO	69.126	-136.6817
PROSOPE	St.1	Westerlies	MEDI	5	0.020	-0.056	0.00	0.004	21.66	36.71	0.13	aboveDCM	13	OLIGO	36.09	-5.1975
	St.1	Westerlies	MEDI	30	0.020	-0.007	0.029	0.040	16.70	36.70	0.54	aboveDCM	13	OLIGO	36.09	-5.1975
	St.1	Westerlies	MEDI	80	0.200	0.759	0.177	2.466	14.86	37.11	0.34	belowDCM	13	MESO	36.09	-5.1975
	UPW	Coastal	CNRY	5	0.216	1.900	0.062	NA	16.30	36.11	2.82	inDCM	24	OLIGO	31.02	-10.0357
	St.MIO	Westerlies	MEDI	5	0.012	0.046	0.004	0.902	26.04	38.58	0.02	aboveDCM	160	OLIGO	33.9923	22.0152
	St.MIO	Westerlies	MEDI	50	0.014	0.014	0.000	0.829	17.14	37.94	0.11	aboveDCM	160	OLIGO	33.9923	22.0152
	St.MIO	Westerlies	MEDI	90	0.016	0.012	0.004	0.557	15.19	38.47	0.19	inDCM	160	OLIGO	33.9923	22.0152
	St.9	Westerlies	MEDI	65	0.135	0.765	-0.001	1.652	13.93	38.12	0.29	belowDCM	89	OLIGO	41.8849	10.4834
	St.DYF	Westerlies	MEDI	15	0.007	0.007	0.008	1.401	22.30	38.32	0.10	aboveDCM	22	OLIGO	43.4236	7.8533
	St.DYF	Westerlies	MEDI	50	0.054	1.124	0.004	2.800	13.67	38.37	0.43	inDCM	22	OLIGO	43.4236	7.8533

Table S.1. Some features of the sampling stations. DCM: Deep Chlorophyll Maximum, NA: not determined, OLIGO: Oligotrophic status, MESO: Mesotrophic status, EUT: Eutrophic status. Latitude and longitude are expressed in decimal degrees. Oceanic biomes and oceanic provinces were defined according to Longhurst (2007): Oceanic biomes: polar, westerly winds (westerlies) and coastal boundary (coastal) domains. Oceanic provinces: SARC = Atlantic sub-Arctic; ARCT = Atlantic Arctic; BPRL = Boreal polar; MEDI = Mediterranean Sea; PSAE = Eastern Pacific subarctic gyres; CNRY = Canary current coast.

		Clone libraries		
Dataset	Station	N° sequences	N° OTUs	Coverage
ARCTIC	Z01	90	17	92
	Z07	39	9	95
	Z11	44	10	91
	Z18	18	8	94
	M09	30	10	83
	Z68	39	10	85
	Z65	37	10	89
	Z61	19	9	79
	Z59	24	14	58
BOUM	St.1	4	4	-
	St.21	11	6	-
	St.A	109	8	98
	St.C	84	15	93
MALINA	ARC1	50	5	96
	PAC2	18	1	100
	St.170	26	1	100
	St.360	55	11	96
	St.398	30	4	90
	St.620	60	9	95
	St.680	62	7	95
	St.694	23	6	78
	St.697	41	20	63
PROSOPE	St.1	122	24	90
	UPW	45	18	78
	St.MIO	134	23	95
	St.9	25	11	68
	St.DYF	67	14	93

Table S.2. Properties of the distribution of *pufM* sequences at each sampling station. Coverage was calculated as follows: $C_x = 1-(n_x/N)$ where n_x is the number of clone types that are encountered only once in library x and N is the total number of clone analyzed.

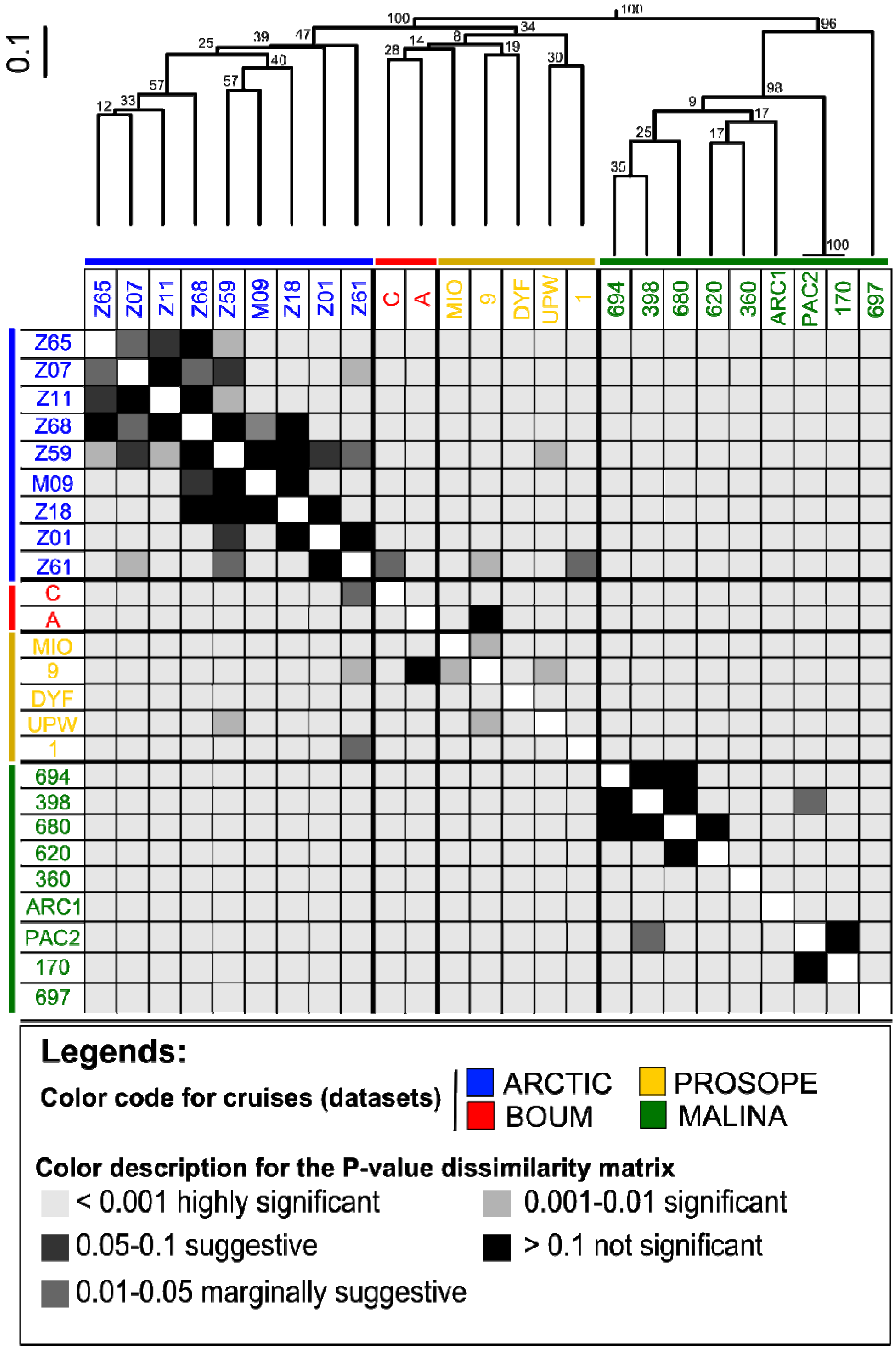

Figure S.1: Hierarchical clustering of samples based on phylogenetic similarity between AAP bacterial communities and dissimilatory matrix showing the significance of p-values**.** Values on nodes represent confidence values of clusters (Jackknife counts, 100 resamplings). [Note that stations St.1 and St.21 from BOUM dataset were excluded from the analysis because of their low number of sequences (see Table S2)].

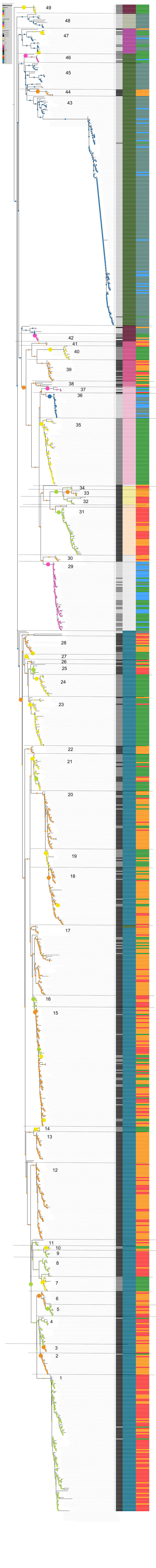
49
48
47
46
45
44
43
42
41
40
39
38
37
36
35
34
33
32
31
30
29
28
27
26
25
24
23
22
21
20
19
18
17
16
15
14
13
12
11
10
9
8
7
6
5
4
3
2
1
